# Global Research Trends and Hotspots in the Role of Cholesterol in Colorectal Cancer: A Bibliometric Analysis

**DOI:** 10.1155/humu/6546114

**Published:** 2025-05-24

**Authors:** Jun Pu, Yamin Zhao, Yu Wu, Tianqi Wu, Yue Ben, Yuting Sun, Jue Gu

**Affiliations:** ^1^Affiliated Hospital of Nantong University, Medical School of Nantong University, Nantong, China; ^2^Nantong Second People's Hospital, Nantong, China; ^3^Nantong Tumor Hospital & Affiliated Tumor Hospital of Nantong University, Nantong, China; ^4^Civil Aviation Shanghai Hospital, Gubei Branch of Ruijin Hospital Affiliated to Shanghai Jiao Tong University School of Medicine, Shanghai, China

**Keywords:** bibliometrics, cholesterol, colorectal cancer, hotspots, trend

## Abstract

**Background:** Cholesterol metabolism is important in colorectal cancer (CRC) pathogenesis, influencing tumorigenesis and therapeutic responses. Despite extensive research, fragmented insights and a lack of systematic analysis hinder the identification of global trends, collaboration networks, and emerging hotspots in this rapidly evolving field.

**Methods:** A bibliometric analysis of 1532 publications (2003–2024) from the Web of Science Core Collection was conducted using VOSviewer, CiteSpace, and R software. Metrics included publication trends, country/institution contributions, author networks, journal influence, keyword co-occurrence, and citation bursts.

**Results:** Global publications exhibited exponential growth, peaking at 114 in 2024. The United States (347 articles, 17,613 citations) and China (316 articles) dominated research output, yet institutional collaboration remained limited. Key journals included *PLOS One* and *Cancer Research*. Keyword evolution shifted from “physical activity” and “carcinogenesis” to “cholesterol metabolism,” “gut microbiota,” and “nanomedicine.” Statins showed preventive potential but raised concerns with prolonged use. Mechanistic insights highlighted nanoparticle-driven therapies as promising strategies to enhance chemosensitivity and reverse immunosuppression.

**Conclusion:** This inaugural comprehensive bibliometric analysis delineates the current research landscape of cholesterol in CRC, with particular emphasis on elucidating metabolic reprogramming mechanisms and fostering cross-disciplinary convergence. Future priorities include validating statins' efficacy via randomized trials, leveraging multiomics for personalized therapies, and fostering global collaboration to address geographic disparities and translational challenges.

## 1. Introduction

The role of cholesterol in the pathogenesis of colorectal cancer (CRC) has garnered increasing scientific attention due to its dual impact on tumor development and therapeutic potential [1–4]. As a leading cause of cancer-related mortality worldwide, CRC remains a significant public health challenge, with metabolic dysregulation emerging as a key risk factor [5, 6]. Studies have shown a correlation between serum lipid profiles and the use of cholesterol-lowering drugs with an increased risk of CRC in the UK Biobank cohort [7]. The production of branched-chain amino acids (BCAAs) by *Bacteroides fragilis* has been found to promote CRC tumorigenesis by modulating host cholesterol metabolism [8]. Additionally, a novel hypoxia-responsive circular RNA, circINSIG1, has been identified as upregulated in CRC tissues, correlating with advanced clinical stages and poor survival outcomes. This circRNA promotes the ubiquitination of the key cholesterol metabolic regulator INSIG1, thereby inducing cholesterol biosynthesis and facilitating CRC proliferation and metastasis [9]. Targeting the cholesterol–RORalpha/*γ* axis to degrade c-myc has been shown to inhibit CRC progression [10]. Furthermore, the compound methyl-*β*-cyclodextrin has been reported to suppress tumor growth by targeting colon cancer cells and tumor-associated macrophages, providing new treatment ideas [11]. The immune checkpoint molecule B7H3 increases ferroptosis resistance in CRC by inhibiting cholesterol metabolism [12]. Moreover, the SREBP1/FASN/cholesterol axis has been implicated in promoting radioresistance in CRC. Collectively, these studies highlight the multifaceted regulatory roles of cholesterol metabolism in CRC development, metastasis, treatment response, and prognosis [13–15]. Despite extensive research on the role of cholesterol metabolism in CRC progression, existing studies tend to be fragmented, lacking a systematic and comprehensive analysis of global research trends, collaboration networks, and emerging hot topics. This knowledge gap hinders the identification of interdisciplinary opportunities in this rapidly evolving field.

Bibliometric analysis is a powerful and objective method that employs quantitative techniques to assess and analyze scientific literature. It provides valuable insights into research outputs, citation patterns, collaboration networks, and emerging trends within specific research areas [16]. A bibliometric analysis of cholesterol and CRC-related literature can help map the current research landscape; identify the most influential countries, institutions, authors, and journals in the field; and reveal the most prominent research areas of interest. By analyzing citation bursts and keyword trends, this approach can also predict future directions in research. Therefore, the primary objective of this study is to conduct a comprehensive bibliometric analysis of publications related to cholesterol and CRC, retrieved from the Web of Science Core Collection (WoSCC) database. Through this analysis, we aim to address existing knowledge gaps, provide an overview of the current research status, and offer actionable insights for researchers in the field of CRC and cholesterol metabolism. This not only facilitates a deeper understanding of the historical development and current state of research but also guides future research endeavors and fosters global partnerships in precision oncology and metabolic intervention strategies.

## 2. Materials and Methods

### 2.1. Data Source and Search Strategy

**Figure 1:**
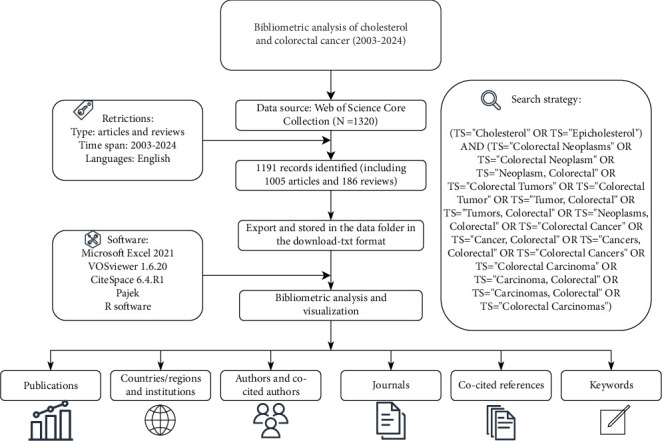
Flow diagram of the retrieval strategy and analysis.

The WoSCC provides the most comprehensive and highest-quality evidence. The plain text format downloaded from WoSCC can be directly used for visualization in software such as VOSviewer and CiteSpace. We retrieved cholesterol and CRC publications from the WoSCC database, covering the period from January 1, 2003, to December 31, 2024. The search strategy is illustrated in the flowchart in Figure 1. At least two researchers independently conducted the literature search and inclusion process to minimize bias in the literature retrieval, data inclusion, and duplicate removal processes. In case of discrepancies, a third researcher was consulted for a final decision. Additionally, all included publications were retrieved from the database on the same day.

### 2.2. Inclusion and Exclusion Criteria

The following criteria were used to select publications for inclusion in the analysis: (1) articles and reviews related to cholesterol and CRC, (2) publications written in English, and (3) publications published between 2003 and 2024. Exclusion criteria included the following: publications unrelated to cholesterol and CRC, non-English publications, types other than articles and reviews, and those published before 2003. In the end, 1532 papers were chosen for bibliometric analysis.

### 2.3. Data Visual Software and Statistical Analysis

After gathering all data from the WoSCC database, we carefully reviewed and cleaned the dataset by eliminating duplicates and correcting spelling errors to ensure smooth compatibility with subsequent software processing and analysis. To analyze and visualize the data, we employed a range of bibliometric and visualization tools, including Microsoft Excel 2021, R software, VOSviewer 1.6.20, and CiteSpace 6.4.R1. VOSviewer and CiteSpace were then used for bibliometric analysis and visualization, covering aspects such as countries/regions, authors, institutions, journals, keywords, and references. In the VOSviewer, the total link strength of a node reflects the number of connections it has, with higher values indicating greater collaboration. In CiteSpace, centrality measures a node's significance, with larger nodes and purple outer rings denoting higher importance. The Kleinberg algorithm in CiteSpace detects citation bursts, identifying keywords with substantial frequency changes over time. This functionality allows CiteSpace to highlight emerging research trends, shifts in focus, and potential future directions in the field. Through the Dimensions platform (https://app.dimensions.ai/discover/publication), we employed DOI-based matching to extract corresponding Altmetric Attention Scores (AASs) for each publication, subsequently creating data visualizations to assess social media dissemination impact as a supplementary measure to conventional bibliometric evaluation metrics.

## 3. Results

### 3.1. Trends in Publication Growth

**Figure 2:**
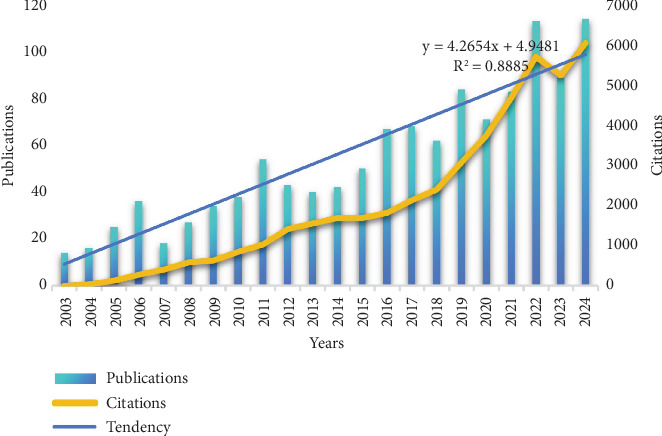
The global trend of annual publications and citations related to cholesterol and CRC from 2003 to 2024.

The overall trend in the number of publications on cholesterol and CRC from 2003 to 2024 is shown in Figure 2. From 2003 to 2024, the number of publications fluctuated annually, with the trend line indicating exponential growth. The output from 2003 to 2007 remained relatively low, with publications fluctuating around 25 articles per year. Peak publication years were observed in 2006, 2011, 2016, and 2019. The number of publications reached its peak in 2024, with 114 articles. This finding suggests that research on cholesterol in the context of CRC is gradually becoming a prominent topic. However, due to various influencing factors, the publication counts during the period from 2003 to 2024 exhibited some fluctuations and instability, though the overall trend shows exponential growth.

### 3.2. Countries/Regions and Institutions

**Figure 3:**
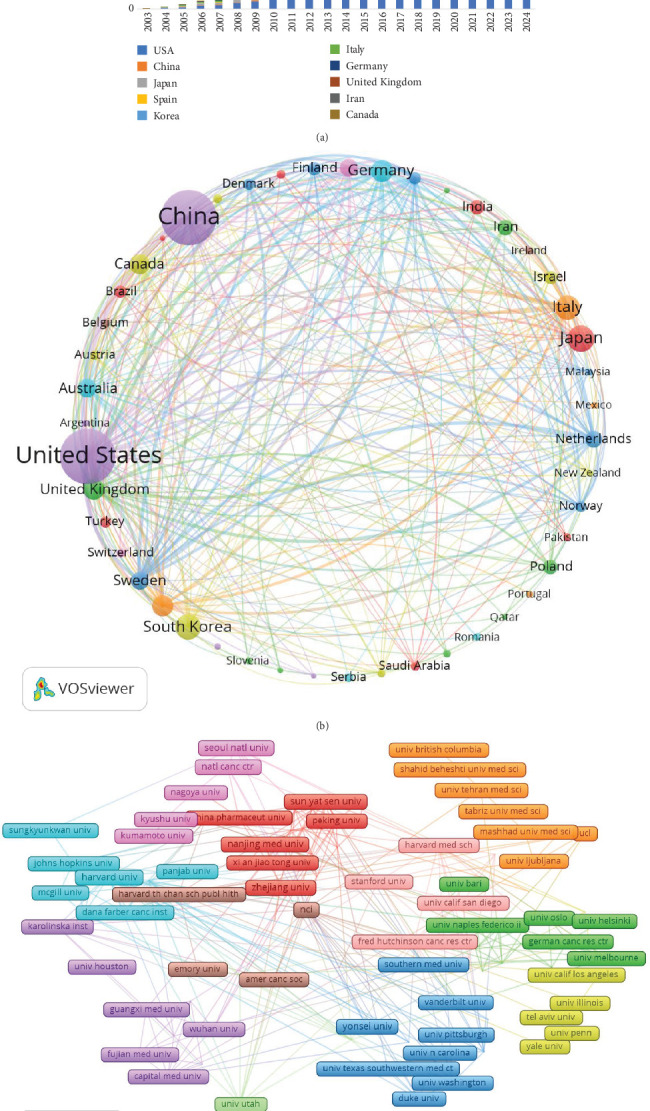
(a) Columnar statistical chart of the number of annual publications in the top 10 countries/regions. (b) A collaborative network of countries/regions involved in cholesterol and CRC research. (c) Coauthorship network of institutions involved in cholesterol and CRC research. The size of the nodes represents the number of publications from each country/region or institution, while the thickness of the lines between nodes indicates the strength of collaboration between them.

Research on cholesterol and CRC has been conducted by researchers from 5044 institutions across 343 countries/regions.  Table 1 ranks the top 10 countries/regions and institutions by productivity. The United States is the primary contributor, with 347 publications, followed by China (316 publications) and Japan (85 publications). In terms of citation count, the United States leads with 17,613 citations, followed by China (6798 citations) and Italy (3378 citations). Although the United States has the highest number of publications, its average citation per paper (*N* = 52.8) is notably lower than that of New Zealand (*N* = 235) and Scotland (*N* = 164.25). Moreover, China and Japan show comparatively lower average citation counts, suggesting that researchers from countries with high publication output must focus on producing higher-quality, more innovative, and widely acknowledged academic papers in their areas of expertise. Furthermore, the significant increase in annual publications from the United States and China in recent years further underscores their leadership in this domain (Figure 3a).

Additionally, we conducted a network mapping analysis to identify and illustrate the collaborative relationships between countries/regions (Figure 3b). The size of the nodes reflects the strength of connections between countries/regions, with a centrality score above 0.1 signifying stronger ties and higher relevance. With a centrality of 0.65, the United States plays a crucial role as a key connector, facilitating collaboration and paper exchange among nations. Moreover, China (*N* = 0.12), Italy (*N* = 0.14), and England (*N* = 0.15) are also significant nodes in the cluster, representing their importance in international collaboration.

A total of 5044 institutions have engaged in research investigating the relationship between cholesterol and CRC (Figure 3c). Analysis revealed Harvard University (53 publications), Harvard University Medical Affiliates (37 publications), Brigham and Women's Hospital (24 publications), and Sun Yat-sen University (23 publications) as leading contributors with the highest publication outputs. Notably, seven of the top 10 ranked institutions are based in the United States, demonstrating a concentrated research focus on cholesterol's role in colorectal carcinogenesis within American academia and underscoring their disciplinary prominence. Citation metrics showed Harvard University as the most frequently cited institution (*N* = 2881), followed by Brigham and Women's Hospital (*N* = 1206) and Sun Yat-sen University (*N* = 388), with two of the top three being US-based. When assessing research impact, the University of Illinois exhibited the highest mean citation count per publication (*N* = 129.1), surpassing Virginia Commonwealth University (N = 118.0) and the University of Houston (*N* = 114.4). Network analysis revealed suboptimal collaboration patterns, with most institutional nodes presenting centrality scores below 0.1. This metric suggests limited international institutional collaboration, emphasizing the need to establish cross-institutional partnerships and multinational consortia to accelerate translational advances in this field.

### 3.3. Author and Coauthor Analysis

**Figure 4:**
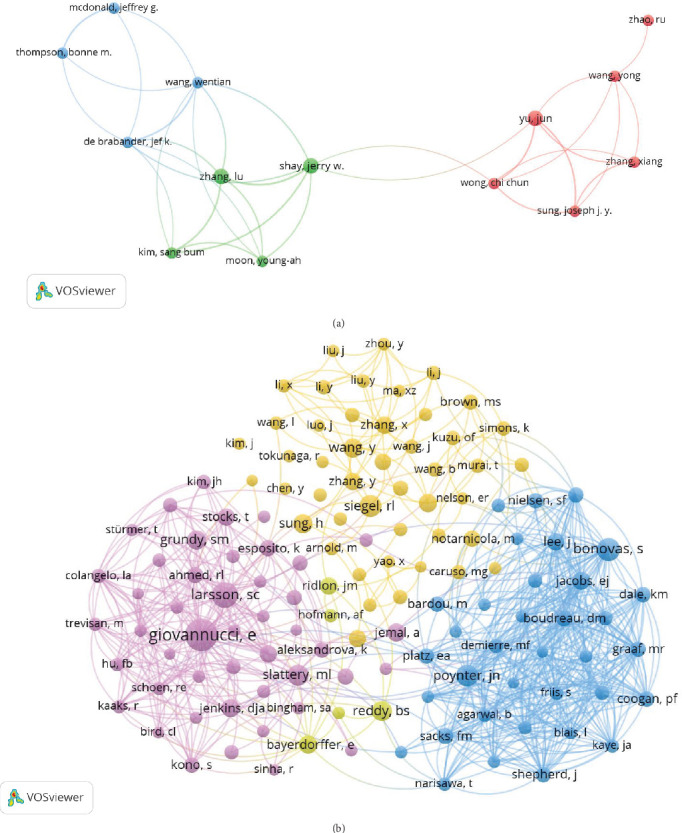
Visualization of (a) author collaborations and (b) coauthorship networks on cholesterol and CRC. In the author and coauthorship collaboration network, the size of each node corresponds to the number of publications by the respective author, while the thickness of the lines between nodes indicates the strength of collaboration between authors.

A total of 8158 authors have contributed to research exploring the role of cholesterol in CRC pathogenesis.  Figure 4, respectively, illustrates collaborative networks between the top 10 authors by *h*-index and the most cocited authors in cholesterol-related CRC and leukemia research, with node colors representing distinct research clusters and connecting lines indicating collaborative relationships.  Table 2 further delineates the top 10 most productive and highly cited authors in cholesterol and CRC studies. Riboli Elio demonstrated the highest *h*-index (10) with 632 citations, followed by Boeing Heiner, Palmqvist Richard, and Tumino Rosario, each achieving an *h*-index of 9 and citation counts of 697, 552, and 606, respectively. These metrics underscore their pivotal contributions and leadership in elucidating cholesterol's mechanistic roles in colorectal carcinogenesis. Cocitation analysis revealed critical knowledge foundations: Giovannucci E emerged as the most influential cocited author (citation count = 182, link strength = 1482), followed by Larsson SC (citations = 115, link strength = 707) and Bonovas S (citations = 89, link strength = 1294). Their seminal studies have substantially advanced theoretical frameworks and experimental paradigms in this domain.

### 3.4. Journals and Cited Academic Journals

**Figure 5:**
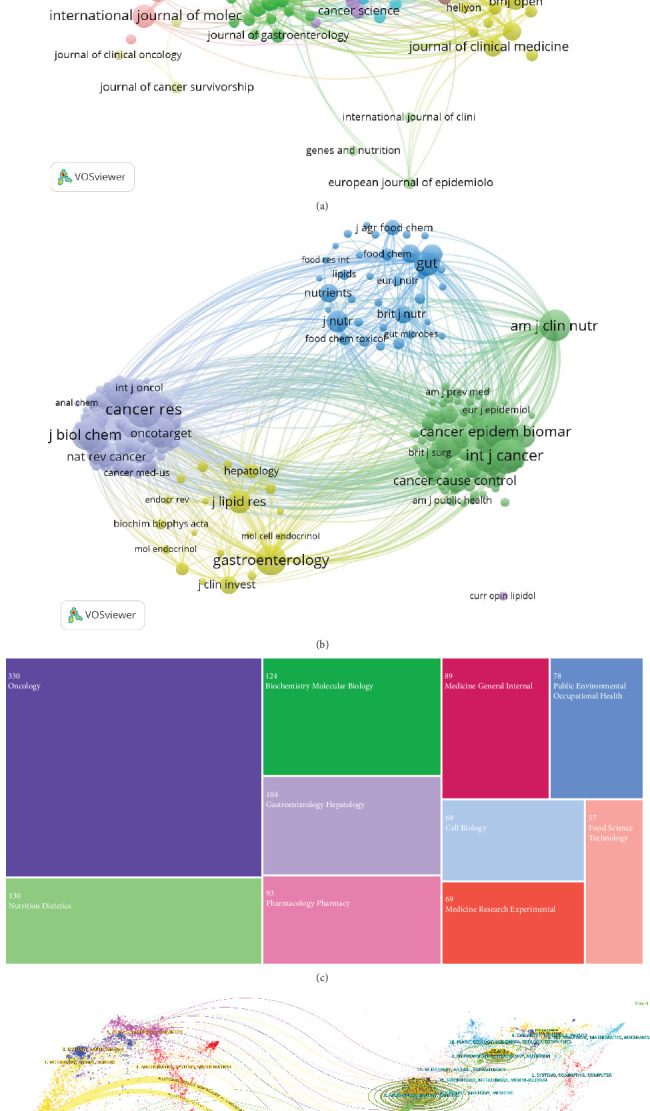
(a) Network visualization of cholesterol and CRC research journals. (b) Cocitation network visualization of cholesterol and CRC research journals. The size of the nodes indicates the frequency of citations, and the lines between the two nodes represent citations made by a single journal. (c) Distribution of literature research topics. (d) CiteSpace dual-map overlay view depicting the cocitation relationship between cholesterol and CRC research journals. Journals on the right are the cited ones, those on the left are the citing journals, and the colored paths in the center represent the cocitation relationships.

A total of 561 academic journals have actively published research on the role of cholesterol in CRC.  Table 3 ranks the top 10 journals related to cholesterol's role in CRC based on the number of published articles and citations. The journal with the highest number of publications is *PLOS One* (*N* = 20), followed by *Cancers* (*N* = 19) and *International Journal of Molecular Sciences* (*N* = 14). Among the top 10 journals, six are categorized as Q1 in the Journal Citation Reports (JCR 2024) and two as Q2, and four articles have an impact factor greater than 4. The top 10 journals by citation frequency include *Cancer Research* (*N* = 1198), followed by the *International Journal of Cancer* (*N* = 1050) and *The American Journal of Clinical Nutrition* (*N* = 938). Of these, nine journals are classified as Q1 and one journal as Q2 in JCR, with three journals having an impact factor greater than 25 and one boasting a remarkable impact factor of 21.71.  Figure 5a displays the top 10 journals by article publication quantity in this field, where the size of the nodes correlates positively with the number of publications—larger nodes indicate greater influence or activity in the field. Journals clustered by the same color exhibit higher thematic relevance. The lines between the journals represent citation or collaboration relationships, with thicker lines indicating stronger associations. *BMC Cancer*, *PLOS One*, and the *International Journal of Cancer* occupy central positions in the clusters and have strong connections with other journals, fostering close collaboration. The cocitation network map of journals (Figure 5b) illustrates the degree of connection between cocited journals. The journals are grouped into four clusters, with the size of the nodes representing the number of connections and the width of the lines indicating the strength of these connections. The purple cluster centered around *Cancer Research* and the yellow cluster centered around *Gastroenterology* are the most prominent, indicating the strongest thematic ties and highest citation counts. Journals within the same color cluster share similar topics, emphasizing their interrelation in research on the role of cholesterol in CRC.

The dual-overlay analysis results highlight the disciplines engaged in cholesterol research within the context of CRC. As illustrated in Figure 5c, research on cholesterol and CRC spans multiple disciplines. The visualization is split into two parts: The left side showcases citing journals, highlighting the latest advancements, while the right side features cited journals, representing the foundational knowledge. The lines connecting these two sections depict the relationships between citations, visualizing the collaborative and knowledge-linkage dynamics between cited and citing journals. These connections reflect the flow of major disciplines within journals, providing insights into the interdisciplinary relationships within this field. The *Z*-score standardizes citation counts, emphasizing stronger connections, smoother trajectories, and higher scores, represented by thicker connecting lines in the visualization. The length of the ellipses indicates the number of authors, while the width indicates the number of publications. As shown in Figure 5c, in the yellow citation path, journals in the fields of molecular biology and immunology typically cite journals in molecular biology and genetics (*Z* = 7.0248075, *f* = 16,146). In the green citation path, journals in medicine, medical, and clinical fields frequently cite journals in molecular biology, genetics, health, nursing, and medicine (*Z* = 4.5904136, *f* = 10,760; *Z* = 3.206434, *f* = 7698).  Figure 5d displays the various research topics covered by the literature in this field, including oncology (*N* = 330), nutrition dietetics (*N* = 130), and biochemistry molecular biology (*N* = 124), among 10 other topics. The results are consistent with those in Figure 5c, highlighting the interdisciplinary approach embedded in the research on cholesterol and CRC.

### 3.5. Literature Cocitations Analysis

Table 4 presents the 10 most-cited articles on the role of cholesterol in CRC, with the most cited being the study by Poynter JN et al., titled “Statins and the Risk of CRC” (*N* = 75). This study discusses how statins, effective lipid-lowering drugs, may inhibit colon cancer cell growth. Several secondary analyses of clinical trials suggest that statins can reduce the risk of CRC. A population-based case-control study in northern Israel, involving structured interviews and prescription record verification, found that individuals who used statins for at least 5 years had a significantly lower relative risk of CRC. After adjusting for factors such as aspirin use, physical activity, and hypercholesterolemia, the association remained significant. Furthermore, the use of fibrates was not linked to a reduced CRC risk. Therefore, statin use was associated with a 47% relative reduction in CRC risk, although the absolute risk reduction may be low, warranting further investigation into its overall preventive benefits.

**Figure 6:**
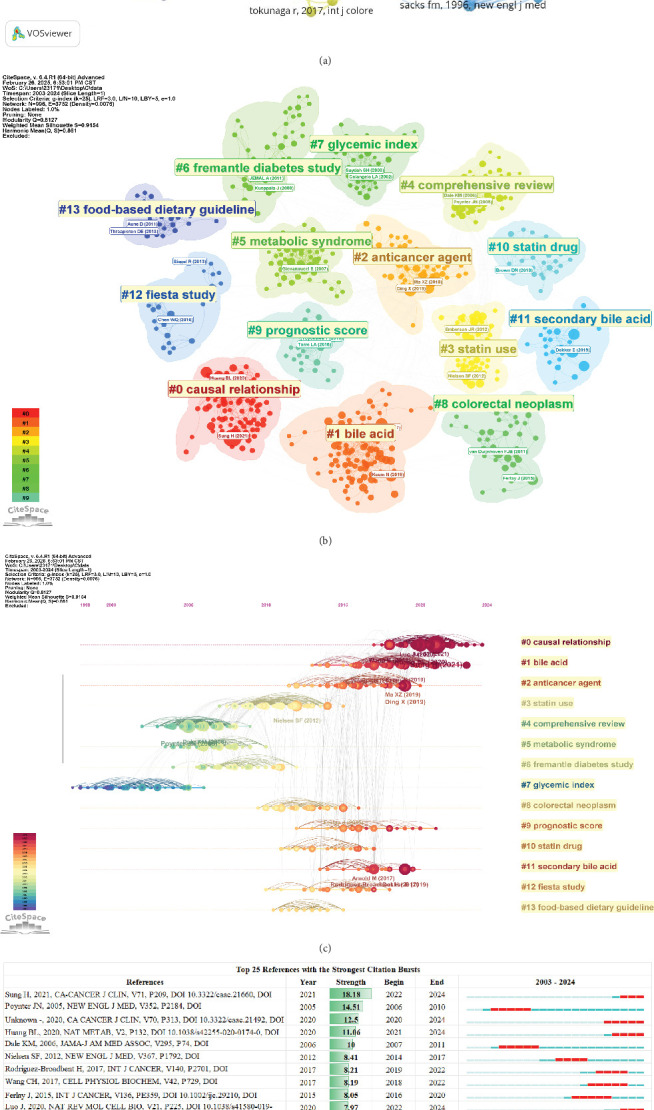
(a) Literature cocitations related to cholesterol and CRC. The size of each node indicates the frequency of citations, and the lines connecting two nodes represent references that are cited together in the same paper. (b) Literature cocitation clustering. (c) Publishing timeline of the literature. Each node represents different documents, with larger nodes indicating higher citation frequency, and the lines connecting them represent documents that are cocited. (d) The top 25 references with the strongest burst intensity were generated by CiteSpace. Blue bars represent the time intervals, while red bars indicate the burst periods. The start and end years of the bursts are also displayed.

A synthesis of the literature reveals the research progress on cholesterol's role in CRC, as shown in Figure 6, which identifies 120 key nodes. It is noteworthy that the majority of widely cited articles appeared between 2000 and 2016, underscoring the rapid development and significant achievements in this field during this period. Figure 6 presents a cocitation cluster analysis of the literature on cholesterol's role in CRC, revealing the thematic categorization within the field. The diagram displays 15 clusters, with the first labeled as “#0 casual relationship,” the second as “#1 bile acid,” and the third as “#2 anticancer agent.” These clusters correspond to popular topics in the various stages of CRC research in the context of leukemia.

Figure 6c shows a timeline view of the clusters formed by cocited literature. Nodes arranged along the same line represent a single cluster, each labeled on the right side. Larger nodes indicate higher citation frequency, suggesting that nodes on the left typically correspond to classic or relatively outdated topics, while nodes on the right represent newer emerging themes. The connections between nodes reflect the citation relationships between the studies. Some of the emerging research hotspots include “#1 casual relationship,” “#2 bile acid,” “#3 anticancer agent,” and “9 prognostic score.”

To visually represent trending topics, we created Figure 6d, where the year lines correspond to the period from 2003 to 2024, with the red line indicating the duration of citation bursts. Notably, the article by Hyuna Sung, “Global Cancer Statistics 2020: GLOBOCAN Estimates of Incidence and Mortality Worldwide for 36 Cancers in 185 Countries” (2022–2024, burst strength 18.18), has the highest citation burst intensity. This study suggests that by 2040, the global cancer burden will reach 28.4 million cases, a 47% increase from 2020, with the rise in risk factors associated with globalization and economic growth potentially exacerbating this trend. Additionally, the paper by Binlu Huang, “Cholesterol Metabolism in Cancer: Mechanisms and Therapeutic Opportunities” (2021–2024, burst strength 11.06), argues that intracellular and extracellular cues in the tumor microenvironment reprogram cholesterol metabolism, thereby promoting tumorigenesis. Cholesterol-derived metabolites play a complex role in supporting cancer progression and suppressing immune responses. Preclinical and clinical studies indicate that manipulating cholesterol metabolism can inhibit tumor growth, reshape the immune landscape, and rejuvenate antitumor immunity. The paper discusses therapeutic strategies aimed at disrupting cholesterol metabolism and how combining this approach with existing cancer therapies could produce synergistic effects, providing new therapeutic opportunities [17].

**Figure 7:**
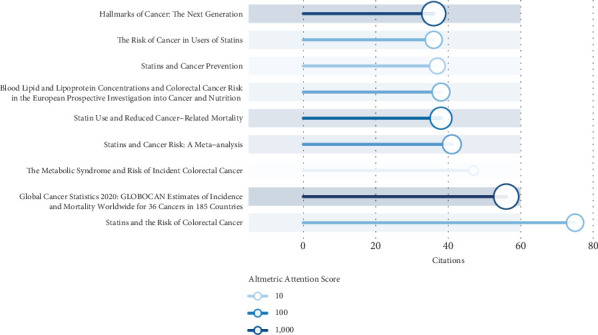
Visualization of the AAS for the top 10 most-cited articles.

The AAS, as a weighted composite measure of all online attention garnered by a scholarly publication, has emerged as a promising metric for assessing the societal impact of research—an increasingly crucial dimension in today's digitally connected and social media–dominated academic landscape [18].  Figure 7 presents a comparative analysis of citation counts and AASs for this field's top 10 most-cited publications. Notably, the article entitled “Global Cancer Statistics 2020: GLOBOCAN Estimates of Incidence and Mortality Worldwide for 36 Cancers in 185 Countries” achieved the highest AAS, indicative of its extensive dissemination and remarkable engagement across social media platforms. This was closely followed by “Hallmarks of Cancer: The Next Generation” and “Statin Use and Reduced Cancer-Related Mortality” in terms of their Altmetric performance.

### 3.6. Keywords Analysis

**Figure 8:**
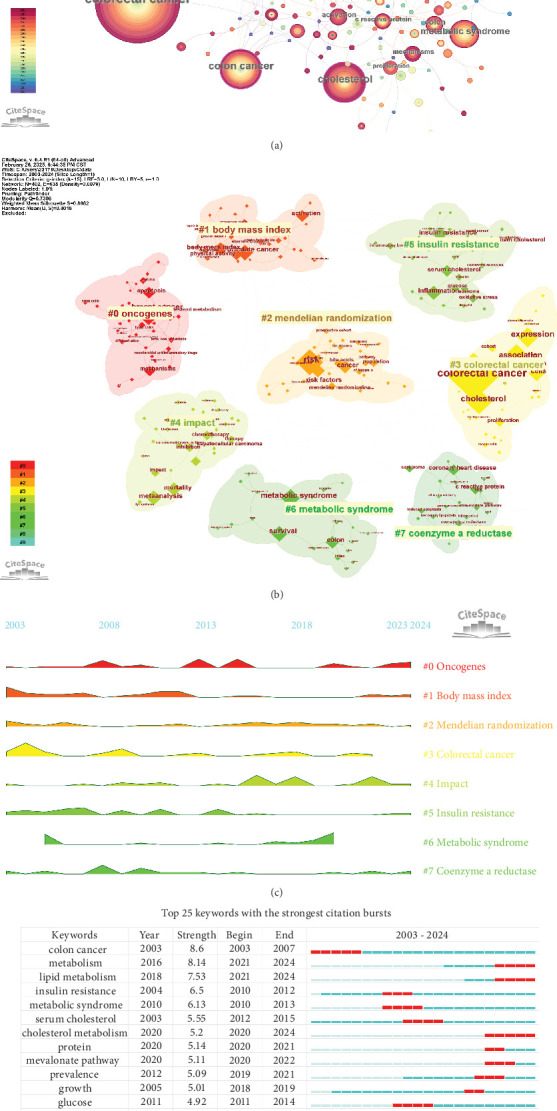
Network map of keywords in cholesterol and CRC research. (a) Keyword co-occurrence network drawn by CiteSpace. The size of the nodes indicates the frequency of keyword occurrences, while the lines connecting the nodes represent the co-occurrence frequency between keywords. The outer purple circle represents a centrality greater than 0.1, which is a popular keyword in this field. (b) The top eight keywords clustering in CiteSpace. The labels correspond to the names of the clusters formed based on keyword similarities. (c) Time peak graph of keyword cluster. The uplifted area map represents that the research keyword cluster was widely used during this period. (d) The top 25 keywords with the strongest citation burst intensities.

A total of 5449 keywords were extracted from 1191 papers.  Figure 8a illustrates the visualization analysis of these keywords. After removing noninformative terms, the most frequent keyword was “risk” (199 occurrences), followed by “expression” (120 occurrences), “metabolic syndrome” (91 occurrences), and “survival” (71 occurrences). The keyword clustering analysis provides deeper insights into the thematic distribution of cholesterol research in the context of CRC, enhancing the clarity of specific research topics within this field.  Figure 8b shows the clustering visualization of the keyword network, displaying the top eight clusters.

Cluster 0 primarily consists of studies related to the mechanisms of CRC development, including keywords such as “oncogenes,” “lipid rafts,” “statins,” and “cancer stem cells.” Cluster 1 focuses on factors related to the prevention and incidence of CRC, including “body mass index,” “physical activity,” “growth factor I,” “plasma glucose,” and “dietary recommendations.” Cluster 2 explores various research directions related to CRC and cholesterol, with keywords including “Mendelian randomization,” “colorectal polyps,” “LDL cholesterol,” and “bile acids.” Cluster 3 centers around studies on tumor cell apoptosis, incorporating terms like “immune checkpoints,” “transcription factor,” “membrane raft,” and “antitumor.” Cluster 4 is dedicated to CRC prognosis and risk factor assessment, including the establishment of prognostic models, with keywords such as “CRC prognosis,” “16S rRNA gene amplicon sequencing,” “gut bacteria,” “postmenopausal osteoporosis,” “Conut score,” and “nanomedicine.” Cluster 5 highlights dietary recommendations for the prevention of CRC and risk–benefit assessments, with keywords such as “dairy consumption,” “nutrigenomics,” and “cigarette smoking.” Cluster 6 is related to metabolic syndrome, with key terms like “apolipoprotein A-I,” “PCSK9,” “particle size,” and “abdominal obesity.” Cluster 7 focuses on lipid metabolism, including terms such as “coenzyme A reductase,” “low-density lipoprotein,” “all-cause mortality,” and “C-reactive protein.” Keyword clustering helps clarify the multifaceted research prospects regarding cholesterol's role in CRC, highlighting active research areas and emerging topics.

Figure 8c depicts the peak diagram of keyword clustering, where higher peaks indicate stronger keyword bursts during specific periods, aiding our understanding of field development. In 2003, Cluster 1's “body mass index” and “Mendelian randomization” were major research topics. Around 2013, “oncogenes” in Cluster 1 and “insulin resistance” in Cluster 5 gained widespread attention. In recent years, “oncogenes” and “body mass index” have remained central to the research focus.

Analysis of keyword bursts provides insights into the popularity and usage of keywords over time. As shown in Figure 8d, the top 25 keywords with the strongest bursts are displayed. In the earlier period, heavily cited keywords included “carcinogenesis,” “insulin resistance,” and “metabolic syndrome.” In later years, research on cholesterol related to CRC shifted toward mechanism studies, with key areas such as “lipid metabolism,” “protein,” “cholesterol metabolism,” “mevalonate pathway,” and “gut microbiota” emerging as focal points. This temporal analysis highlights the ongoing evolution of research on the role of cholesterol in CRC and reflects the changing focus and emerging areas of interest over time.

## 4. Discussion

### 4.1. General Information

**Figure 9:**
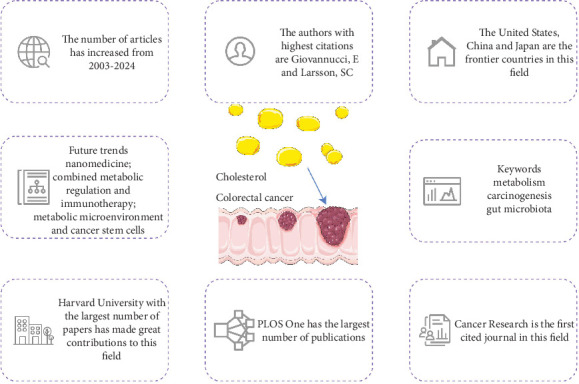
Visualization conclusion after bibliometric analysis.

The multifaceted role of cholesterol metabolism in CRC has garnered increasing scientific attention in recent years. Despite extensive investigations into cholesterol-related pathways in CRC, a systematic bibliometric synthesis of this burgeoning field remains absent, leaving critical research gaps and emerging trends underexplored. To address this knowledge void, we conducted the first comprehensive bibliometric analysis using WoSCC data to delineate current research landscapes and forecast future trajectories. Our analysis of 1191 rigorously selected publications revealed significant growth patterns and highlighted contributions from diverse nations and institutions, aiming to provide actionable insights for researchers. The main conclusions of this paper are shown in Figure 9.

Global publications exhibited a consistent upward trajectory, peaking in 2024 with 114 articles—the highest annual output to date. While publication volumes displayed minor fluctuations over the past two decades, annual counts stabilized near 100 in recent years, underscoring sustained interest in cholesterol and CRC interactions. The phase of gradual growth from 2003 to 2016 laid essential groundwork for translational research, while the 2022 output surge likely reflects advancements in understanding lipid metabolism and cancer biology interfaces. This upward trend persists despite periodic variations, driven by evolving collaborations, funding availability, and technological innovations.

Notable disparities emerged in research productivity and impact. The United States dominated both publication volume (*N* = 347) and citation counts (*N* = 17,613), cementing its leadership in cholesterol and CRC research. China demonstrated substantial output (*N* = 316) with moderate citation impact (*N* = 6789), suggesting potential opportunities for enhancing research quality and innovation. Network mapping revealed robust international collaborations, with the United States exhibiting the highest centrality (0.65), followed by China, Italy, Germany, and Spain. However, limited cross-institutional cooperation and resource-sharing mechanisms highlight the need for strategic partnership frameworks.

The authorship landscape revealed challenges in sustained research engagement, with most of the contributors publishing 3–5 articles. While influential researchers like Giovannucci E, Larsson SC, and Bonovas S drove seminal work, the limited depth of collaborative networks underscores the necessity for structured interdisciplinary initiatives. Enhanced mentorship programs and incentivized collaborations between lipidologists, oncologists, and immunologists could accelerate breakthroughs in cholesterol and CRC mechanisms.

*PLOS One* emerged as the most prolific journal, followed by *Cancers* and the *International Journal of Molecular Sciences*. Notably, 3/5 top productive journals and 9/10 highly cited journals ranked in JCR Q1, confirming their academic authority. The strong representation of high-impact journals reflects researchers' strategic alignment with platforms that maximize clinical translation potential. Emerging intersections with nanomedicine and advanced therapeutic modalities suggest promising frontiers for industrial innovation.

Dual-map overlay analysis demonstrated substantial cross-pollination between molecular biology, immunology, and clinical medicine. Publications originating from molecular biology domains exhibited direct translational impacts on immunotherapy development and clinical practice guidelines, emphasizing the interconnected nature of cholesterol and CRC research.

The majority of publications exhibit a weak correlation between citation counts (20–80) and AAS (10–1000), suggesting that traditional academic impact (citations) and societal engagement (AAS) may be governed by distinct mechanisms. High-AAS publications are predominantly characterized by themes such as cancer epidemiology reviews and groundbreaking basic research, which are more likely to attract attention on social media, possibly due to their broad applicability or transformative nature. Additionally, multiple studies focusing on the association between statins and cancer (e.g., “Statin Use and Reduced Cancer-Related Mortality”) appear in this dataset, indicating sustained interest in this topic among both academic and public audiences.

### 4.2. Foundational Stage

This bibliometric analysis maps the temporal evolution of research focuses and examines how scientific priorities align with CRC pathogenesis and clinical needs. The observed keyword bursts between 2003 and 2009—such as physical activity, dietary fiber, and statins—reflect the field's response to CRC's epidemiological drivers and therapeutic considerations. First, the emphasis on dietary factors like fiber and lifestyle choices such as exercise and sleep health stems from strong evidence linking high body mass index and sedentary behavior to increased CRC incidence [19–22]. High-fat diets, in particular, disrupt bile acid metabolism and promote chronic inflammation, which accelerates carcinogenesis [23]. Second, the focus on statins aligns with their dual role: As cholesterol-lowering agents, they mitigate lipid-driven oncogenic pathways, while their anti-inflammatory effects target metabolic syndrome, a key risk factor for CRC [24, 25]. This analysis also demonstrates how mechanistic research evolved from early observational studies. For instance, statins transitioned from being associated with epidemiological findings to potential targeted therapies [26, 27]. By grounding these trends in biological plausibility, our investigation reveals that research on cholesterol dysregulation in colorectal oncogenesis has evolved from observational risk factor delineation to mechanistic exploration of metabolic reprogramming, mirroring the discipline's paradigm shift from population-level epidemiological characterization to molecularly targeted precision oncology frameworks.

The dysregulation of cholesterol homeostasis is a recognized hallmark of cancer, associated with metastasis and chemotherapy resistance, which are two major causes of cancer-related death. While high serum total cholesterol (TC) and triglycerides are associated with an increased risk of CRC, high-density lipoprotein cholesterol (HDL-C) exhibits a protective effect [28]. Higher HDL-C levels were significantly associated with improved OS in patients with CRC (*p* = 0.004) [29, 30]. Elevated cholesterol levels promote bile acid synthesis, leading to the production of secondary bile acids that induce epithelial cell damage and inflammation [31]. However, recent studies have revealed a paradoxical inverse correlation between TC levels and CRC incidence, which may be attributed to the role of cholesterol in maintaining cell membrane stability and signaling pathways [31]. Cholesterol metabolism plays a multidimensional role in the occurrence and development of CRC by regulating signaling pathways, inflammatory microenvironment, and flora homeostasis [32, 33]. Drugs and dietary strategies targeting this pathway provide new ideas for the prevention and treatment of CRC [34, 35].

Physical activity reduces CRC risk by modulating insulin resistance, gut microbiota, and bile acid metabolism. Prospective studies suggest that engaging in at least 150 min of moderate exercise per week can reduce CRC risk by 15%–25%, especially in postmenopausal women [31]. Dietary fiber, particularly from whole grains and vegetables, exerts a protective effect by generating short-chain fatty acids, inducing tumor cell apoptosis, and inhibiting histone deacetylation [36]. Daily intake of ≥ 25 g of fiber is associated with an 18% reduction in CRC risk [37]. The gut microbiome influences cholesterol metabolism by converting primary bile acids into secondary bile acids [31]. Symbiotic *Candida* is a bacterial driver of colorectal tumorigenesis and may be a potential target for the prediction, prevention, and treatment of CRC [8]. With the increase of LDL-C level, intestinal flora can regulate the function of immune cells and gene expression in the tumor microenvironment, affect cancer-related pathways, and promote CRC progression [33]. LDL-C and its related intestinal flora can provide noninvasive markers for clinical evaluation and treatment of CRC patients [33]. Pathogenic bacteria, such as *Lactobacillus acidophilus*, promote the production of carcinogenic metabolites, while fiber-rich diets enhance butyrate production. Advances in metabolomics and gut microbiome analysis have enabled the development of customized dietary recommendations. For instance, patients with hyperlipidemia may benefit from a combination of statins and fiber to optimize cholesterol metabolism and reduce CRC risk [36].

Precision oncology has also explored targeting cholesterol biosynthesis in tumors harboring carcinogenic KRAS mutations, which rely on de novo cholesterol synthesis for survival [38]. Statins, inhibitors of HMG-CoA reductase, have shown promise in CRC prevention through lipid-lowering and nonlipid-lowering effects, including anti-inflammatory and proapoptotic activities [38]. Observational studies have reported a 15%–20% reduction in CRC mortality among long-term users [31]. However, some studies have found an 85% increased risk of proximal colon cancer with prolonged statin use (> 15 years), challenging the chemoprevention hypothesis [39]. The underlying molecular mechanisms may involve gut microbiota dysbiosis and bile acid axis disruption. Studies reveal that *Clostridium* species producing BCAAs may promote colorectal tumorigenesis by modulating host cholesterol metabolism. Specifically, BCAA-producing commensal *Clostridium* enhances cellular cholesterol biosynthesis through BCAA secretion, which sequentially activates Sonic hedgehog signaling via Smoothened-dependent pathways [8]. Fusobacterium nucleatum exacerbates CRC progression by activating Toll-Like Receptor 4 (TLR4)/nuclear factor-*κ*B (NF-*κ*B) signaling and upregulating microRNA-21 expression, thereby increasing CRC cell proliferation and tumor burden in murine models [40]. Concurrently, aberrant accumulation of secondary bile acids, including deoxycholic acid and lithocholic acid, has been shown to promote CRC through DNA oxidative damage induction, chronic inflammation, and apoptosis resistance [41–44]. Furthermore, statins may induce mitochondrial dysfunction, leading to intracellular reactive oxygen species accumulation that drives mitochondrial and nuclear DNA mutagenesis, potentially contributing to carcinogenesis [45]. Further randomized controlled trials and translational studies are needed to confirm their clinical impact. Emerging strategies targeting cholesterol efflux pathways and lipid raft disruption have made CRC cells more sensitive to chemotherapy [46]. Other approaches, such as disrupting cholesterol-mediated immune suppression, have also gained attention. A proton pump critical for cholesterol uptake in CRC cells reduces tumor-derived Transforming Growth Factor-*β*1 (TGF-*β*1) secretion and restores memory CD8+ T-cell function [47]. Additionally, combination therapies involving cholesterol biosynthesis inhibitors, such as lumacaftor and PD-1 blockade, have demonstrated synergistic antitumor effects by reversing T-cell exhaustion [48]. These findings underscore the potential of metabolic checkpoint inhibitors in enhancing immune therapy responses.

### 4.3. Development Stage

The aforementioned studies suggest that the relationship between cholesterol metabolism and CRC has shifted from macrolevel epidemiological observations to a systematic exploration of molecular mechanisms. With breakthroughs in technologies such as metabolomics and single-cell sequencing [49–51], research from 2010 to 2024 has focused on core pathways driving cholesterol-induced tumorigenesis: the synergistic carcinogenic effects of metabolic syndrome [52], genetic susceptibility revealed by genome-wide association studies [53], the cascade regulation of the mevalonate pathway [54], and innovative applications of nanoparticles (NPs) in targeted delivery [55]. These directions not only elucidate the dynamic interactions between cholesterol metabolism and the tumor microenvironment but also highlight the necessity of cross-scale research, ranging from population-level Mendelian randomization analysis to the regulation of stem cell properties at the cellular level, ultimately extending to the clinical translation of precision delivery systems. It is noteworthy that the deepening of mechanistic research is reshaping treatment paradigms. Our analysis shows that key areas include metabolic syndrome, genome-wide association, NPs, mevalonate pathway, Mendelian randomization, stem cells, and delivery from 2010 to 2024.

Metabolic syndrome, characterized by central obesity, glucose metabolism disorders, and lipid abnormalities, is significantly associated with the progression and poor prognosis of CRC [56–58]. Its carcinogenic mechanisms primarily involve the synergistic effects of systemic chronic inflammation and metabolic dysregulation: On the one hand, chronic inflammation driven by metabolic syndrome promotes epithelial–mesenchymal transition and angiogenesis through activation of the NF-*κ*B signaling pathway. On the other hand, bile acid accumulation resulting from abnormal cholesterol metabolism can disrupt the intestinal barrier function, creating a protumor microenvironment [31]. Recent studies have revealed that gut microbiota dysbiosis and cholesterol metabolites (such as 27-hydroxycholesterol) interact to exacerbate the risk of CRC development in metabolic syndrome patients [59].

At the metastatic mechanism level, abnormal accumulation of free cholesterol can activate hepatic stellate cells through the TLR4/NF-*κ*B/TGF-*β* signaling axis, significantly enhancing the liver metastatic potential of CRC cells [60]. This process is closely linked to increased mortality in patients [61]. Genetic studies have provided new evidence for the connection between metabolic regulation and CRC [62, 63].

The mevalonate pathway, as the core regulatory pathway for cholesterol biosynthesis, plays multiple roles in the occurrence and development of CRC: (1) It promotes tumor cell proliferation through the Ras/ERK signaling axis [64]. (2) Mevalonate kinase weakens the antitumor immune response in microsatellite instability CRC by inhibiting interferon signaling [65]. (3) Inhibition of the mevalonate pathway enhances M1 virus–mediated oncolysis in cancer cells [66]. (4) Recent studies have shown that this pathway is involved in maintaining tumor stem cell properties, suggesting that targeting mevalonate intermediates may disrupt the tumor stem cell niche [67]. Based on these mechanisms, combined lipid metabolism inhibitors with conventional chemotherapy significantly reduce CRC cell viability and allow for chemotherapy dose reduction [68].

In terms of clinical translation, research has confirmed that HDL-C levels and the TC/HDL-C ratio can serve as important prognostic indicators for CRC [30]. Additionally, abnormal changes in bile acids profiles not only provide novel predictive biomarkers for CRC liver metastasis, but their metabolic regulation mechanisms also open up new directions for the development of targeted therapeutic strategies [69]. These advances highlight the central role of metabolic reprogramming in the prevention and treatment of CRC and provide a theoretical basis for the development of personalized treatment plans.

NPs, as novel drug delivery systems, have achieved significant breakthroughs in the treatment of CRC by targeting drug delivery and overcoming tumor chemoresistance, two core mechanisms. Their mechanisms of action primarily involve inducing cell cycle arrest, modulating the immune microenvironment, and enhancing T-cell tumor infiltration capacity [70]. Studies have shown that graphene oxide–based NPs significantly enhance the cytotoxic effects of 5-fluorouracil by specifically disrupting the mitochondrial membrane potential of CRC cells while minimizing systemic toxicity [71]. Similarly, lipid-based NPs conjugated with monoclonal antibodies against Cancer Antigen 125 have enabled site-specific drug release in tumor tissues, improving therapeutic efficacy in preclinical models [72]. Notably, a breakthrough has been made in the development of functionalized gold NP–based integrated diagnostic and therapeutic platforms. By coupling CRC-specific miRNA (e.g., miR-21) aptamers, this system not only serves as a high-sensitivity imaging agent but also allows for real-time monitoring of therapeutic responses, providing an innovative solution for intraoperative margin evaluation and micrometastasis detection [72]. In recent years, cholesterol-based nanoscale delivery systems have achieved a series of breakthrough advancements in CRC treatment. Studies have demonstrated that incorporating cholesterol prodrugs into iterative nanocarriers effectively resolves T-cell exclusion issues, exhibiting superior antitumor efficacy in various CRC animal models [70]. Free cholesterol liposomal formulations delivering CDK12 inhibitors have shown significantly enhanced antitumor activity in CRC therapy [73]. Chen's team innovatively integrated highly immunogenic cholesterol-modified CpG oligonucleotides into autologous *Fusobacterium nucleatum* membrane–based nanovaccines, markedly enhancing antigen presentation efficiency [74]. Notably, novel cholesterol nanocomplexes coloaded with Bcl2 siRNA and quantum dots achieved epidermal growth factor receptor (EGFR)–targeted anticancer effects [75]. Regarding combinational therapeutic strategies, cholesterol-coated NPs loaded with retinoic acid and anti-PD-L1 immune checkpoint inhibitors effectively disrupted the upregulated STAT3/NF-*κ*B pathway crosstalk in CRC [76]. More strikingly, nanosystems composed of an oxaliplatin prodrug coordination polymer core and cholesterol-conjugated SN38 prodrug lipid shell, synergized with immune checkpoint blockade therapy, significantly inhibited tumor growth and invasion in CRC liver metastasis models [77, 78]. In fundamental research, cholesterol butyrate solid lipid NPs effectively suppressed the adhesion and migration of colon cancer cells [79]. Recent advancements have revealed that supramolecular polyrotaxane-based nanotherapeutics can alter cancer cell mechanical properties by depleting membrane cholesterol, significantly enhancing T-cell-mediated anticancer immune responses [80]. Additionally, solid lipid NPs comodified with mitochondria-targeting peptides and EGFR ligands simultaneously disrupted the metabolic supply of fatty acids, cholesterol, and triglycerides, exerting dual inhibitory effects on proliferation and invasion through intensified energy depletion within the tumor microenvironment [81]. In targeted delivery, folate-modified cholesterol liposomal systems successfully facilitated highly selective tumor-targeted delivery of doxorubicin [82]. These innovative studies provide critical theoretical foundations and technical support for the precision treatment of CRC. In the field of antitumor microbiome modulation, novel antibacterial NP vaccines targeting CRC-associated microbiota, specifically *Fusobacterium nucleatum*, have entered preclinical research stages. The vaccine developed by Chen et al. demonstrates remarkable selective preventive and therapeutic effects, eliminating *Fusobacterium nucleatum* without affecting the tumor and intestinal microbiota. It significantly enhances the efficacy of chemotherapy and reduces metastasis in CRC cells infected with *F. nucleatum* [74]. Liposome-encapsulated irinotecan has been FDA-approved for pancreatic cancer and studied in CRC. The liposomal carrier prolongs circulation and preferentially delivers irinotecan/SN-38 to tumors [83]. A recent multicenter Phase II trial evaluated nal-IRI plus 5-FU/leucovorin and bevacizumab as second-line therapy in metastatic CRC. Interim results in 2024 showed an objective response rate of 21.1% and the disease control rate of 84.2% [84]. Median progression-free and overall survival had not been reached at analysis, but the regimen was considered to have great efficacy and controllable safety with no unexpected toxicities [84]. This suggests that nanoliposomal irinotecan can achieve meaningful tumor responses in 5-FU-resistant CRC while maintaining a manageable safety profile. Clinicians have noted that liposomal irinotecan tends to produce less severe neutropenia and diarrhea than standard irinotecan at equivalent doses, likely due to the altered drug release kinetics [83]. NPs exploit the characteristic leakiness of tumor vasculature and can be functionalized with targeting ligands to preferentially concentrate therapeutic agents within colorectal tumors. Examples include EpCAM aptamer–guided gold NPs, which facilitate the targeted delivery of 5-fluorouracil specifically into CRC cells [85], and hyaluronic acid–coated polymeric NPs designed to target CD44 receptors abundantly expressed on colon cancer cells. Both approaches have demonstrated enhanced drug uptake by tumor cells compared to normal tissues.

Several NP-based formulations have achieved superior tumor response or disease control relative to conventional drugs. For example, integration of the camptothecin-based NP formulation, CRLX101, into standard chemoradiation protocols for rectal cancer notably improved complete pathological response rates [86]. Additionally, pegylated irinotecan exhibited prolonged progression-free intervals and substantially extended duration of response in metastatic CRC patients compared with standard irinotecan [87].

Nanocarriers frequently mitigate adverse effects associated with potent chemotherapeutics by modifying biodistribution profiles. Clinical studies consistently indicate that NP-based formulations lower drug-related toxicity without compromising therapeutic efficacy [88]. Liposomal encapsulation of irinotecan, for instance, significantly reduces the incidence of severe diarrhea and neutropenia compared to conventional irinotecan, primarily due to slower drug release kinetics and increased tumor-specific delivery [89]. Similarly, ThermoDox—a thermally sensitive liposomal formulation—selectively releases doxorubicin at the hyperthermic tumor region, potentially minimizing cardiac and systemic toxicities typically observed with free doxorubicin administration [90]. Collectively, these advancements underscore a clear trend whereby NPs enable higher effective drug concentrations at tumor sites while maintaining lower systemic drug exposure, thus sparing normal tissues from off-target effects. These innovative nanomedicine strategies address the three major bottlenecks in CRC treatment—tumor heterogeneity, immune evasion, and dysbiosis—offering new technological pathways for personalized and precision therapy. Many NP therapies show tremendous promise in mice, suggesting that further optimization in delivery and patient selection is needed [91, 92]. As nanomedicine techniques advance, we can expect to see NP-based therapeutics playing an increasingly significant role in CRC management, from metastatic disease treatment to possibly even adjuvant therapy and chemoprevention in high-risk patients.

### 4.4. Trends and Limitations

The future research trends in CRC primarily focus on metabolic pathways, cholesterol metabolism, and the role of the gut microbiota, especially concerning prevention, progression, and treatment. Research highlights the dual role of cholesterol metabolism in CRC and suggests enhancing antitumor immunity by targeting cholesterol synthesis, immune modulation, and metabolic reprogramming. Additionally, dysbiosis of the gut microbiota is closely linked to CRC progression, and modifying the gut microbiota and bile acid metabolism may provide a novel therapeutic approach. Precision medicine, through personalized treatment plans, combined with NP drug delivery systems, metabolic syndrome management, and cholesterol-targeted therapies, presents new strategies for CRC treatment.

This study acknowledges several limitations. First, the analysis, collection, and processing of bibliometric data largely depend on software tools. Although this approach cannot fully replace systematic searches, it allows for a comprehensive analysis of large datasets. Second, this study only collected literature from the WoSCC database, which is a common limitation in bibliometric analysis. This means that some valuable studies by other researchers may have been excluded. However, WoSCC offers broader literature coverage than databases like Scopus, Medline, and PubMed, suggesting that this limitation may not significantly affect the overall trends identified [93]. The data sources for AAS primarily rely on online channels, which may overlook discussions from non-English platforms or niche academic communities, leading to incomplete coverage of global research impact. AAS is highly time-sensitive, with newly published articles potentially receiving high scores due to short-term hot-topic effects, while traditional citations require long-term accumulation. The temporal differences between the two may lead to evaluative bias. A high AAS may reflect public interest or controversy surrounding a particular topic rather than the academic rigor or clinical translational value of the research. Different data providers may apply differentiated weight distribution rules, limiting the comparability of results. The correlation between AAS and traditional academic citations is not yet fully clarified, as high social attention does not necessarily translate into academic impact, and the two may reflect different dimensions of research value.

## 5. Conclusion

This pioneering bibliometric analysis delineates the evolving research landscape of cholesterol's role in CRC from 2003 to 2024, uncovering exponential growth in publications (peak: 114 in 2024) and identifying the United States and China as dominant contributors. Key innovations include the dual role of cholesterol metabolism in CRC: facilitating oncogenesis via lipid rafts while offering immune-modulatory therapeutic targets and the critical interplay between gut microbiota dysbiosis and cholesterol-driven carcinogenesis. Mechanistic insights highlight NP-mediated drug delivery as a novel strategy to enhance chemotherapy sensitivity and reverse immunosuppression. Cross-disciplinary integration, particularly between molecular biology and clinical oncology, underscores the translational potential of metabolic reprogramming. Despite progress, geographic disparities in research impact and limited international collaboration persist. Future priorities include validating statins' chemopreventive efficacy through randomized trials, leveraging multiomics approaches for personalized therapies, and addressing the paradoxical risks of prolonged statin use. This study establishes a roadmap for advancing cholesterol-centric interventions, advocating for global collaboration and precision oncology to mitigate the CRC burden.

## Figures and Tables

**Table 1:** The top 10 countries/regions and institutions in terms of publications in cholesterol and CRC.

**Country**	**Count**	**Centrality**	**Citations**	**Institution**	**Count**	**Centrality**	**Citations**
United States	347	0.65	17613	Harvard University	53	0.14	2881
China	316	0.12	6798	Harvard University Medical Affiliates	37	0.05	188
Japan	85	0.08	2709	Harvard Medical School	32	0.02	329
South Korea	76	0.07	2068	Brigham & Women's Hospital	24	0.05	1206
Italy	71	0.14	3378	Sun Yat Sen University	23	0.02	388
Germany	58	0.11	2473	University of California System	20	0.06	379
Spain	53	0.14	1900	CIBER—Centro de Investigation Biomedica en Red	20	0.09	194
England	50	0.15	1830	Helmholtz Association	19	0.01	371
Canada	45	0.07	2678	Harvard T.H. Chan School of Public Health	19	0.07	143
Australia	41	0.24	1916	National Institutes of Health (NIH), USA	19	0.1	1113

**Table 2:** Top 10 most prolific and cocited authors in cholesterol and CRC.

**Author**	**Citations**	**h** **-index**	**Cited Author**	**Count**	**Total link strength**
Riboli Elio	632	10	Giovannucci, E	182	1482
Boeing Heiner	697	9	Larsson, SC	115	707
Palmqvist Richard	552	9	Bonovas, S	89	1294
Tumino Rosario	605	9	Siegel, RL	82	299
Hallmans Goran	672	8	Poynter, JN	81	917
Kaaks Rudolf	579	8	Slattery, ML	77	441
Overvad Kim	527	8	Reddy, BS	71	342
Tjonneland Anne	533	8	Sung, H	64	247
Vineis Paolo	549	8	Wang, Y	64	410
Bueno-De-Mesquita H. Bas	539	7	Grundy, SM	61	489

**Table 3:** The top 10 journals and cited journals in cholesterol and CRC.

**Journal**	**Count**	**JCR**	**IF**	**Cited journal**	**Count**	**JCR**	**IF**
*PLOS One*	20	Q1	2.9	*Cancer Research*	1198	Q1	12.5
*Cancers*	19	Q1	4.5	*International Journal of Cancer*	1050	Q1	5.7
*International Journal of Molecular Sciences*	14	Q1	4.9	*The American Journal of Clinical Nutrition*	938	Q1	6.5
*Nutrition and Cancer-An International Journal*	13	Q3	2	*Cancer Epidemiology, Biomarkers & Prevention*	908	Q1	3.7
*Anticancer Research*	12	Q4	1.6	*The Journal of Biological Chemistry*	894	Q2	4.0
*Lipids in Health and Disease*	12	Q2	3.9	*PLOS One*	886	Q1	2.9
*BMC Cancer*	11	Q2	3.4	*Gastroenterology*	880	Q1	25.7
*Scientific Reports*	11	Q1	3.8	*The New England Journal of Medicine*	873	Q1	96.2
*Frontiers in Pharmacology*	11	Q1	4.4	*Nature*	832	Q1	50.5
*International Journal of Cancer*	11	Q1	5.7	*Proceedings of the National Academy of Sciences of the United States of America*	713	Q1	9.4

**Table 4:** The top 10 most-cited articles in cholesterol and CRC.

**Author**	**DOI**	**Journal**	**IF**	**JCR**	**Citations**	**Altmetric Attention Score**
Poynter JN	10.1056/nejmoa043792	*The New England Journal of Medicine*	96.2	Q1	75	21
Sung H	10.3322/caac.21660	*CA: A Cancer Journal for Clinicians*	503.1	Q1	56	3615
Ahmed RL	10.1002/cncr.21950	*Cancer*	6.1	Q1	47	3
Dale KM	10.1001/jama.295.1.74	*JAMA: The Journal of the American Medical Association*	63.1	Q1	41	45
Nielsen SF	10.1056/nejmoa1201735	*The New England Journal of Medicine*	96.2	Q1	38	294
Van Duijnhoven FJB	10.1136/gut.2010.225011	*Gut*	23	Q1	38	27
Demierre MF	10.1038/nrc1751	*Nature Reviews Cancer*	72.5	Q1	37	11
Graaf MR	10.1200/jco.2004.02.027	*Journal of Clinical Oncology*	42.1	Q1	36	19
Hanahan D	10.1016/j.cell.2011.02.013	*Cell*	45.5	Q1	36	1530

## Data Availability

Data sharing is not applicable to this article as no new data were created or analyzed in this study.
